# Supplementary material for: Gestational Diabetes-like Fuels Impair Mitochondrial Function and Long-Chain Fatty Acid Uptake in Human Trophoblasts
Source: Int J Mol Sci. 2024 Oct 27;25(21):11534. doi: 10.3390/ijms252111534 (PMC11546831; doi:10.3390/ijms252111534)
Supplement: Supplementary file 1 [file ijms-25-11534-s001.zip › Supplemental Figure Revisions.pdf]

Supplemental Figures

to the manuscript "Gestational Diabetes-like fuels impair mitochondrial function and long chain fatty acid uptake in human trophoblasts"

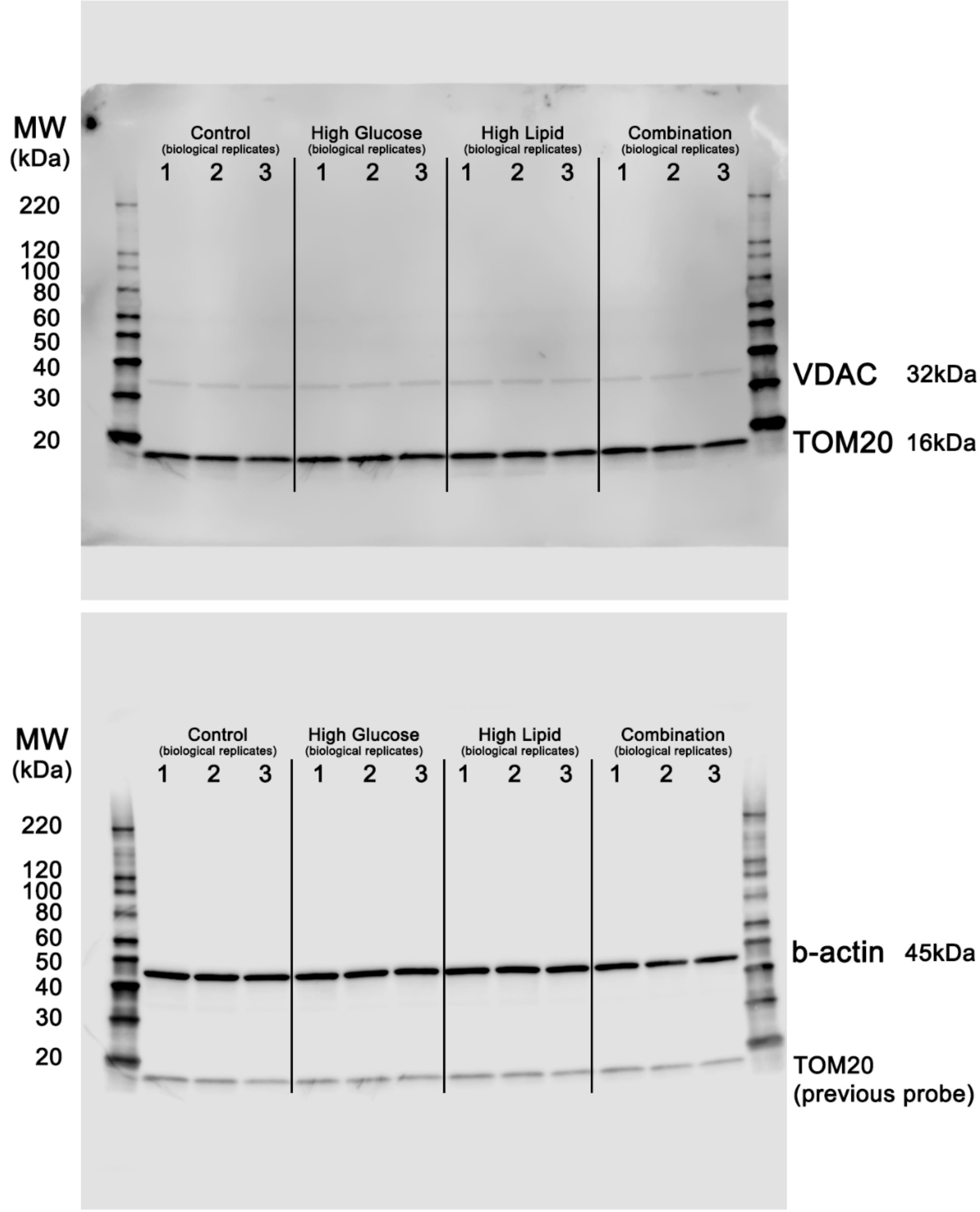

Figure S1. Full representative Western blot of VDAC, TOM20, and beta-actin in BeWo lysates from cells exposed to control, high glucose, high lipid, and combination media. 20 µg of protein was loaded per well. Antibodies applied were Anti-TOM20 (1:1000, Cell Signaling Technology #42406), Anti-VDAC (1:1000, Cell Signaling Technology #4661), Anti-β-actin (HRP-conjugated) (1:1000, Cell Signaling Technology #5125), anti-rabbit IgG-HRP (1:5000, SouthernBiotech #4030-05), anti-mouse IgG-HRP (1:5000, SouthernBiotech #1031-05). Analysis of the content of the abundance from 6 independent biological replicates across two blots generated in parallel.

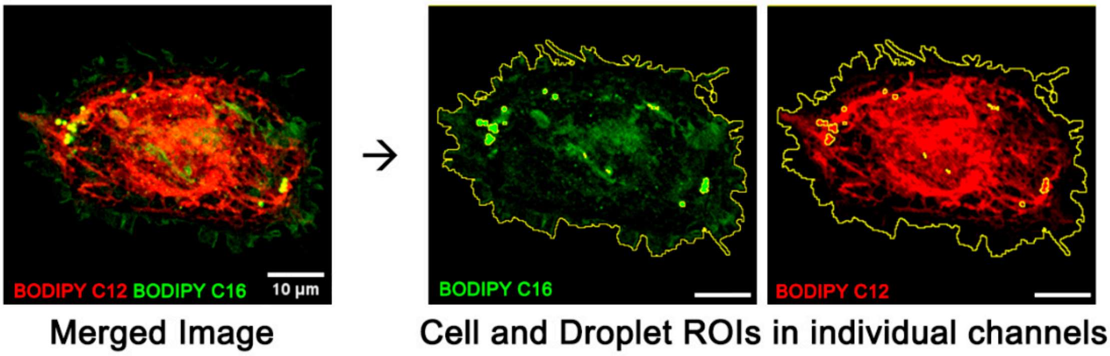

Figure S2. Representative images of droplets were using the green channel fluorescence (BODIPY C16). Droplets were segmented using ImageJ particle analysis with this example of BODIPY C12 imaged at 20 minutes of uptake.

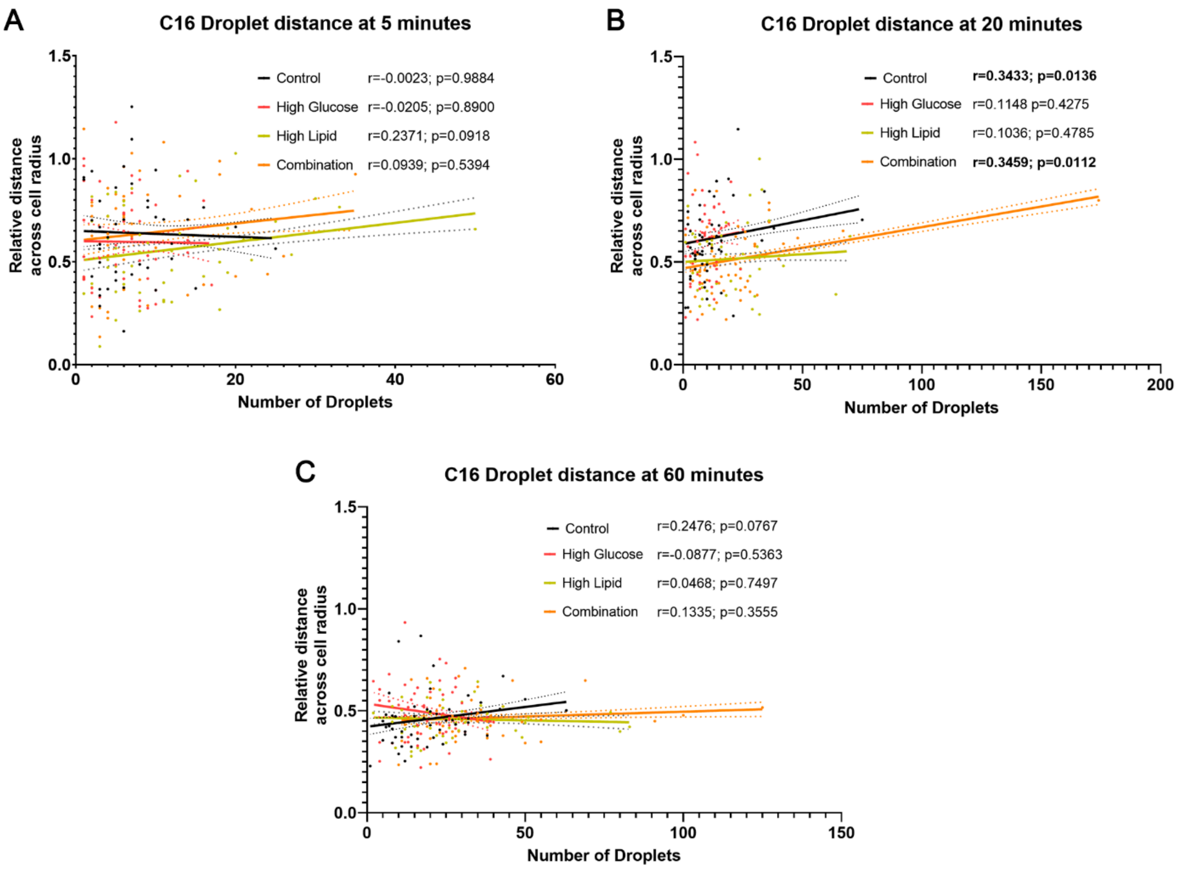

Figure S3. Correlation between realtive distance across cell radius of C16 droplets and number of droplets in each cell at 5 (A), 20 (B), and 60 (C) minutes. Pearson's correlation coefficient (r) was calculated for each exposure group and tested for statistical signficance (**bolded** values = p<0.05), with a simple linear regression +/- 95% confidence interval plotted alongside individual data ponts.

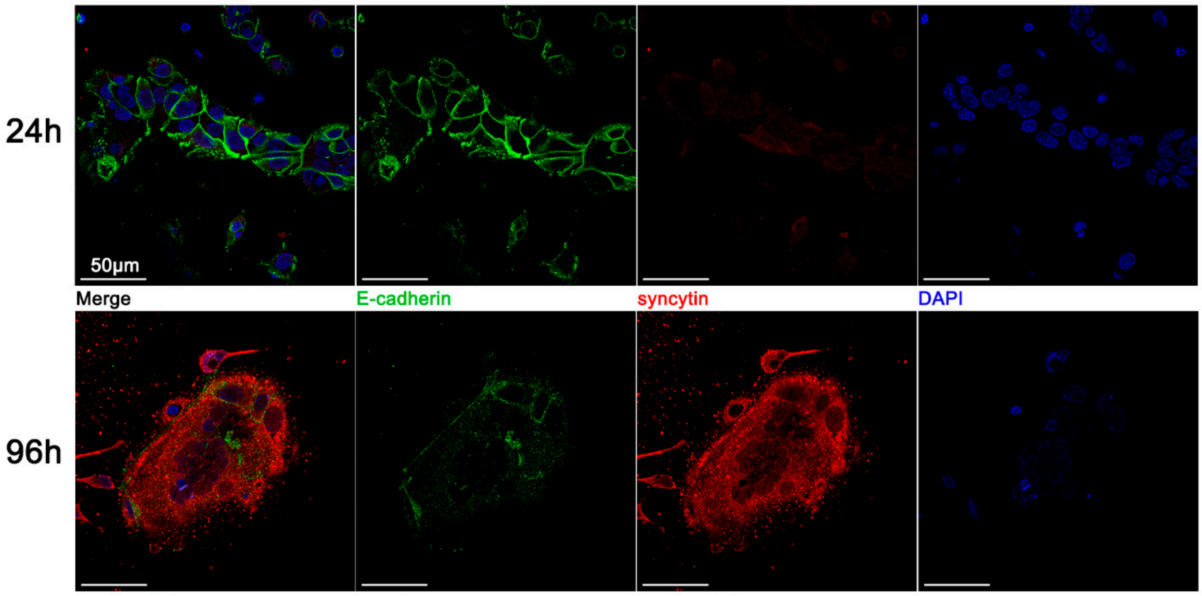

Figure S4. E-cadherin and syncytin abundance over time in primary isolated cytotrophoblasts. Cytotrophoblasts were isolated as described above (Main text - Section 4.2) and cultured for 24 and 96 hours. At these time points, cells were fixed with 4% PFA, permeabilized with 0.3% Triton X-100 in PBS, blocked with 5% normal donkey serum+1% bovine serum albumin (BSA) in PBS-T prior to incubating with primary and secondary antibodies that were diluted in 1% BSA in PBS-T. After antibody incubation and washing with PBS-T, cells were then stained with DAPI for 5 minutes. Dishes were imaged with Nikon A1R confocal microscope at 60x magnfication. Antibodies applied were Anti-ERVW-1 (Syncytin) (Abcam, ab234850) and E-Cadherin (4A2) mouse mAb (Cell Signalling, 14472S).

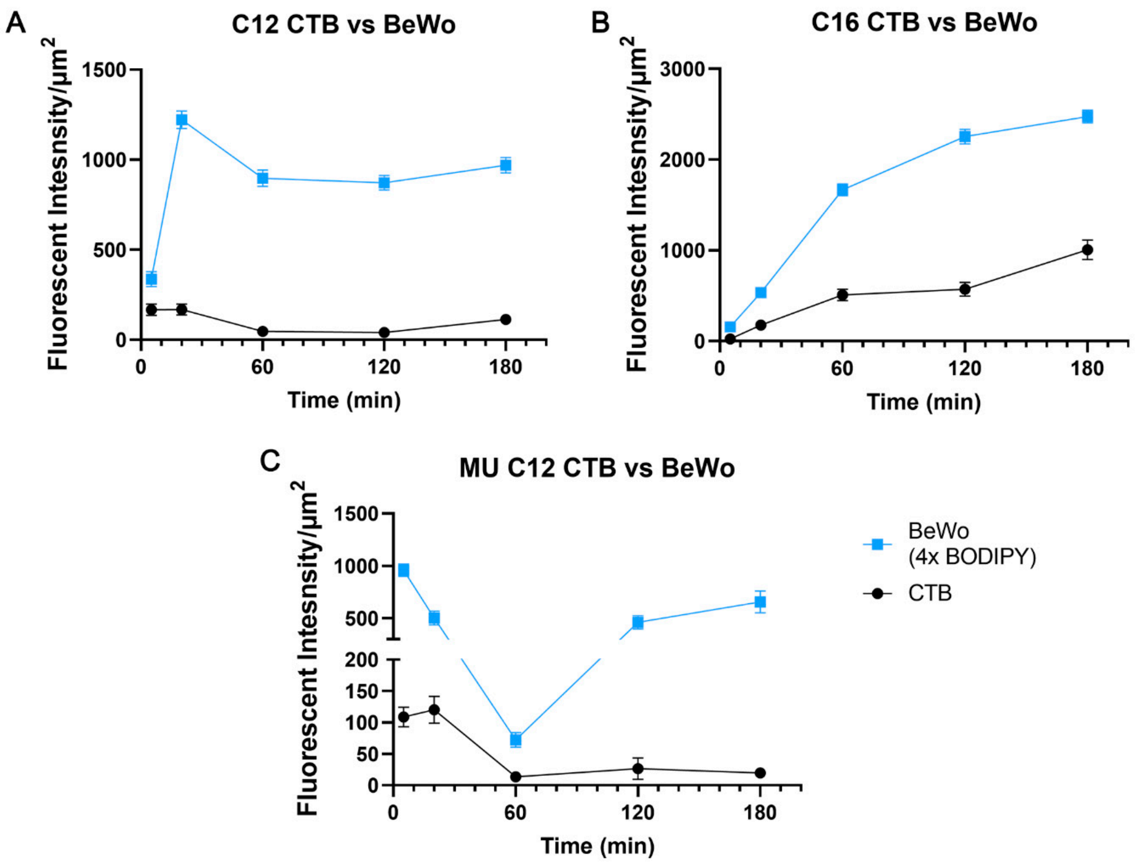

Figure S5. Uptake of BODIPY C12 (A), C16 (B), and MU C12 (C) in non-treated BeWo cells compared to primary isolated CTBs. BeWo cells were exposed to a concentration of BODIPY FAs four times greater than that of CTBs, however, when on the same axes, their rates of uptake can be compared more easily. n=3 Control BeWo uptake trials with 41-55 cells/time point for C12, C16, and MU C12. n=6 patient-derived CTB experiments with 10 cells/time point for C12 and C16 uptake and n=1 patient-derived CTB experiment with 10 cells/time point for MU C12 uptake.

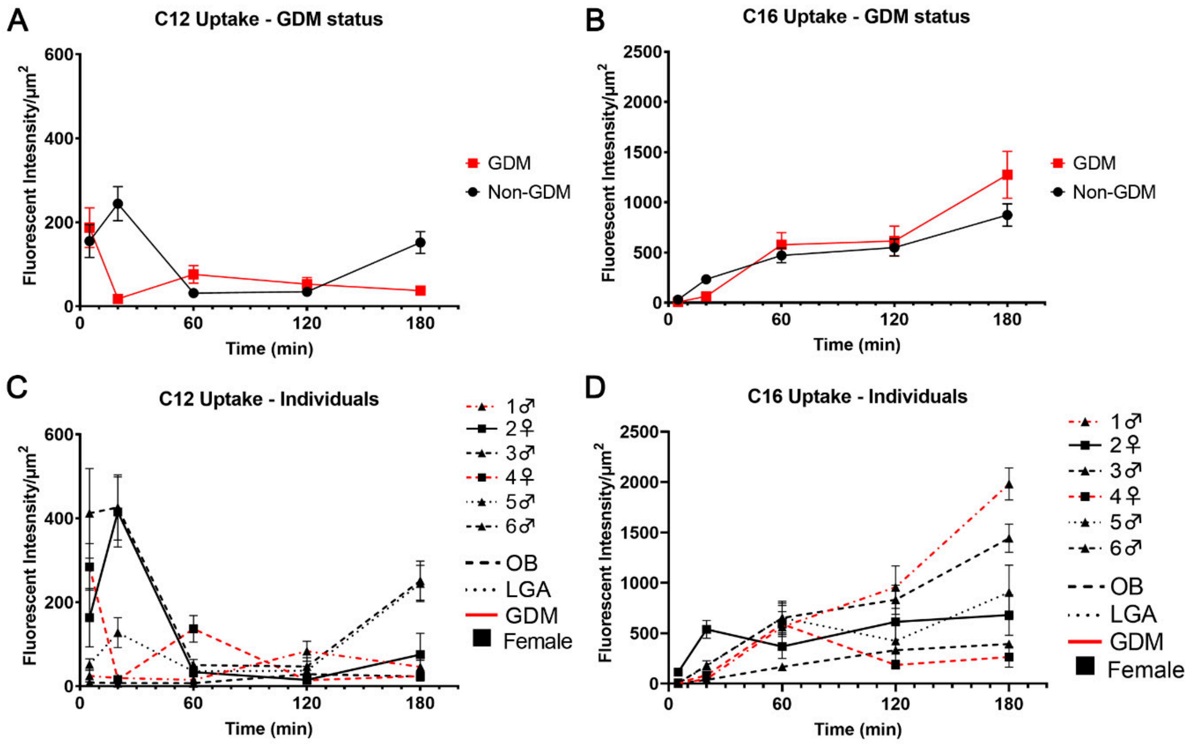

Figure S6. Uptake of BODIPY C12 (A,C) and C16 (B, D) based on patient GDM status (A-B) and based on individual patient (C-D) . There is significant patient diversity is represented by different infant/placenta sex as indicated by data point marker (square = female; triangle = male), color of line indicating GDM status (red=GDM), long dashed lines for obesity status (dashed = BMI >30 kg/m^2), and short dashed lines for LGA status (dashed = LGA). n=6 patients with 10 cells measured/time point/patient. Values are mean +/- SEM.

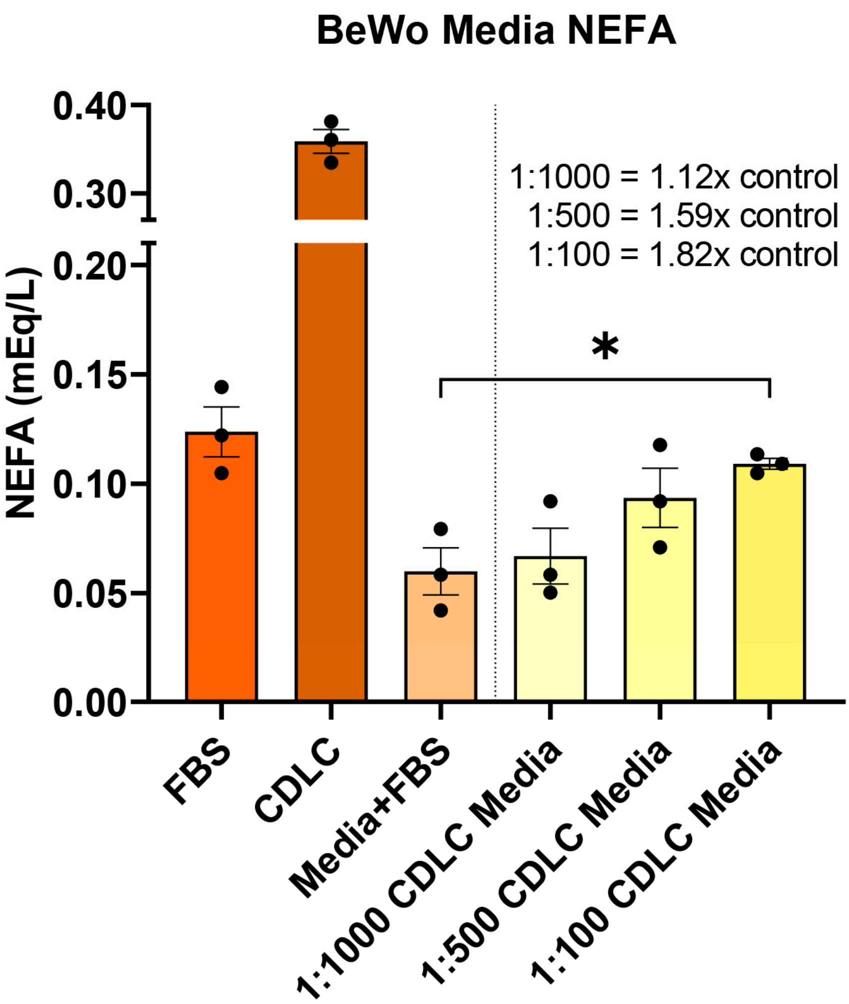

Figure S7. Non-esterified Fatty Acids (NEFA) measured by of BeWo F12K media and lipid supplements used in this study. CDLC supplement ratios were added to F12K media with 10% FBS. Values are data points of replicates (n=3) with bars that represent mean +/- SEM. *p<0.05 by 1way ANOVA with Tukey's multiple comparison test.
