# Supplementary figures and images for: Gestational Diabetes-like Fuels Impair Mitochondrial Function and Long-Chain Fatty Acid Uptake in Human Trophoblasts
Source: Int J Mol Sci. 2024 Oct 27;25(21):11534. doi: 10.3390/ijms252111534 (PMC11546831; doi:10.3390/ijms252111534)

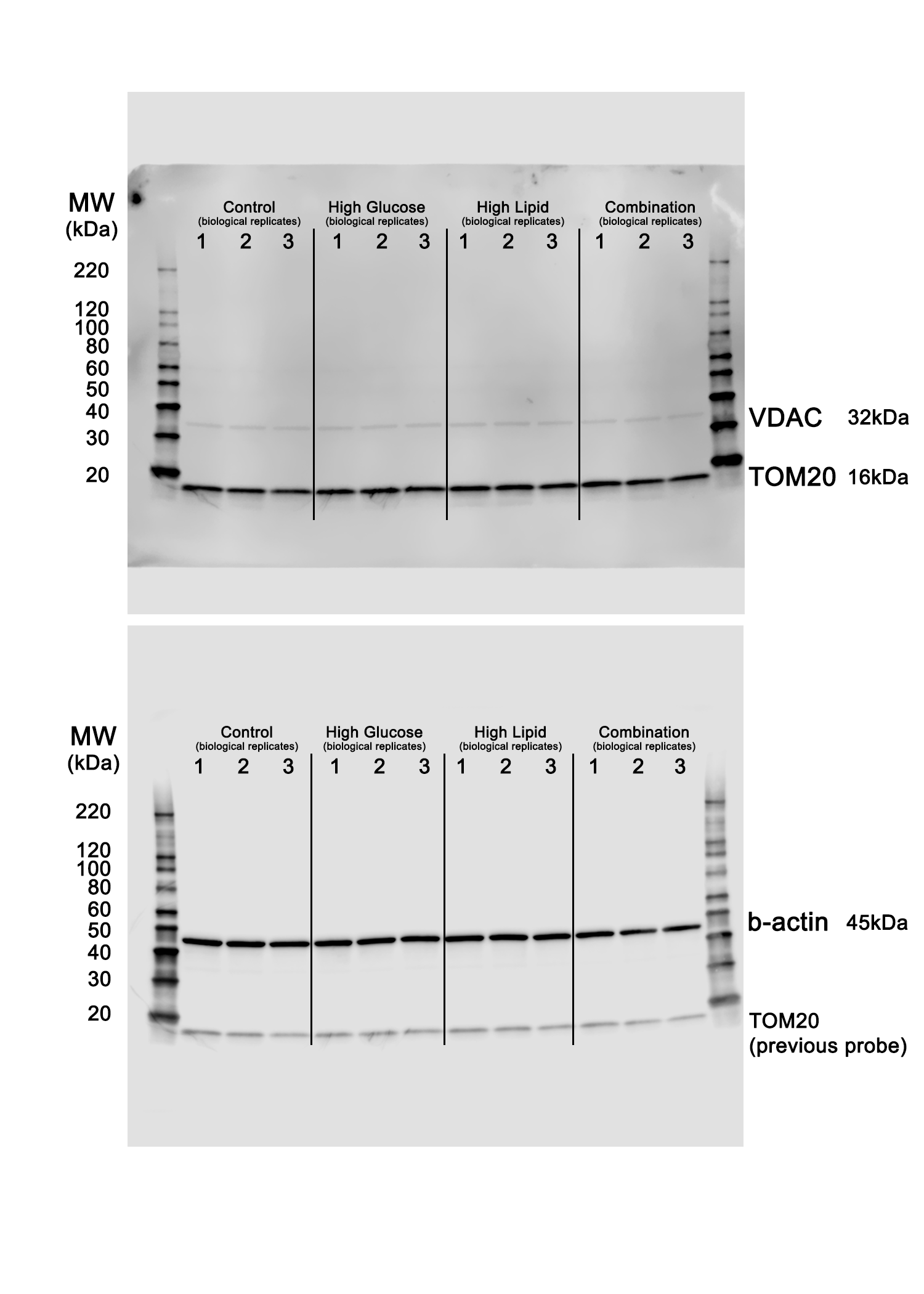

Supplement: Supplementary file 1 [file ijms-25-11534-s001.zip › Figure S1 - Western blots.tif]

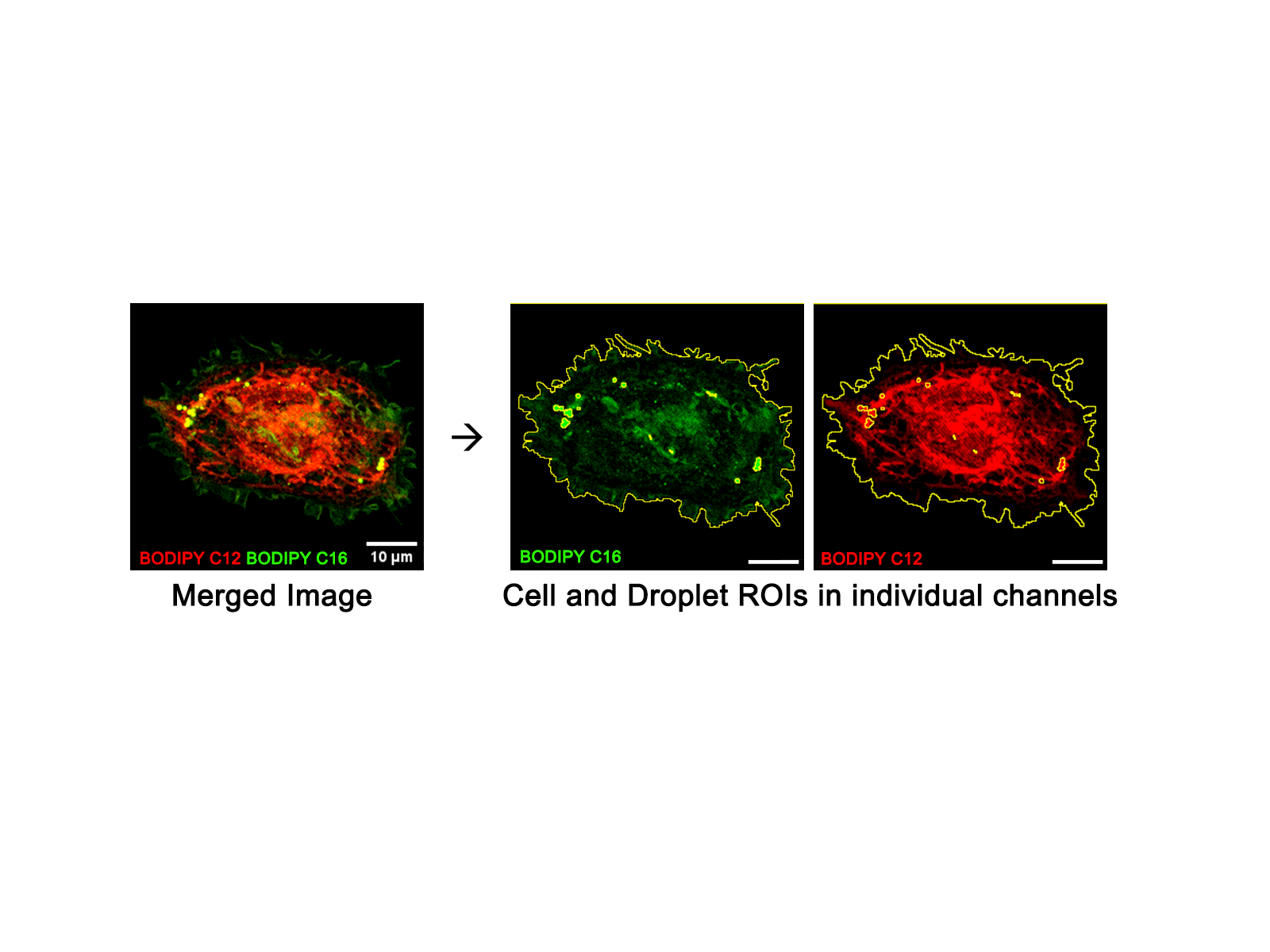

Supplement: Supplementary file 1 [file ijms-25-11534-s001.zip › Figure S2 - Droplet partitioning method.tif]

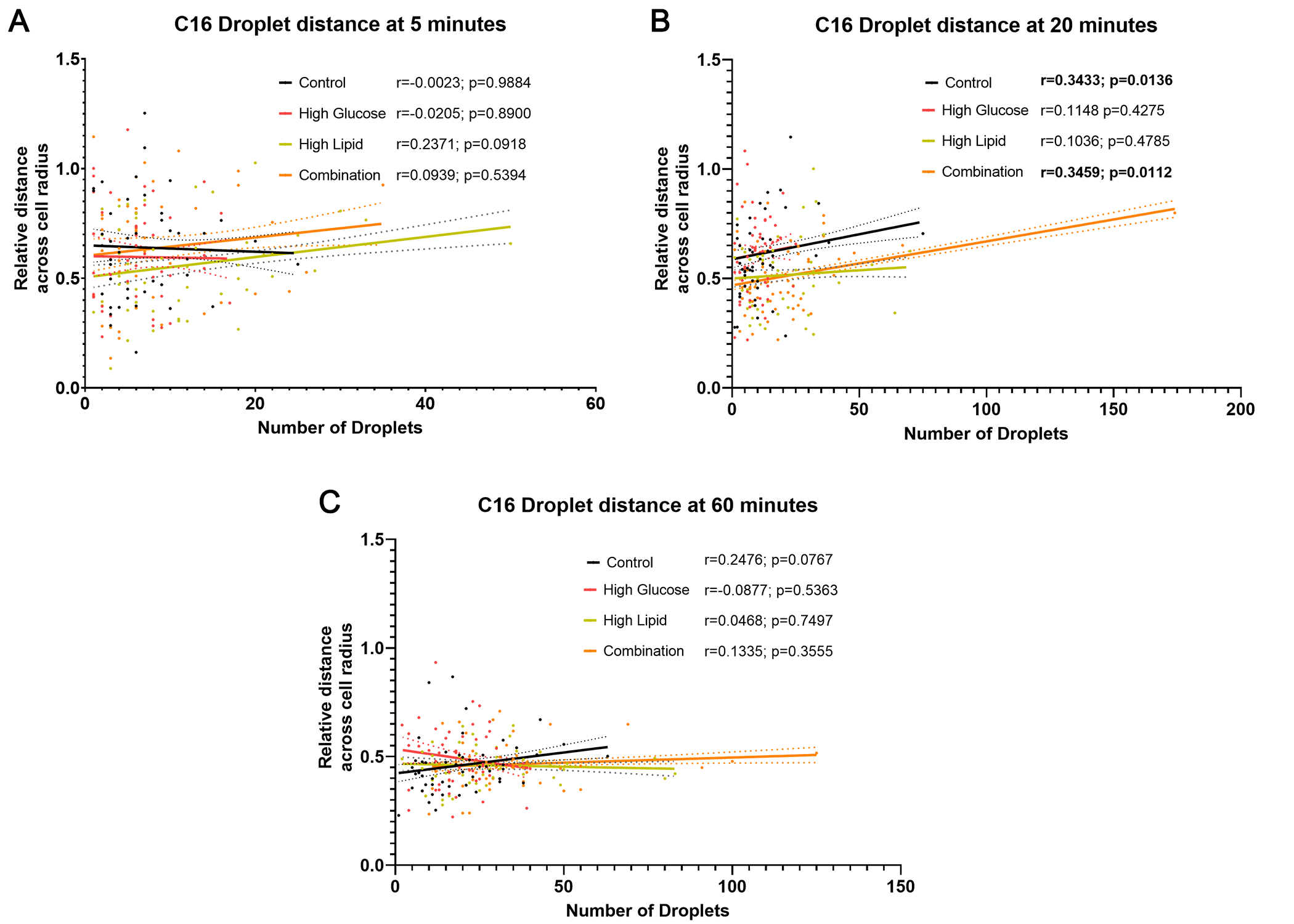

Supplement: Supplementary file 1 [file ijms-25-11534-s001.zip › Figure S3 - Droplet number and distance correlation.tif]

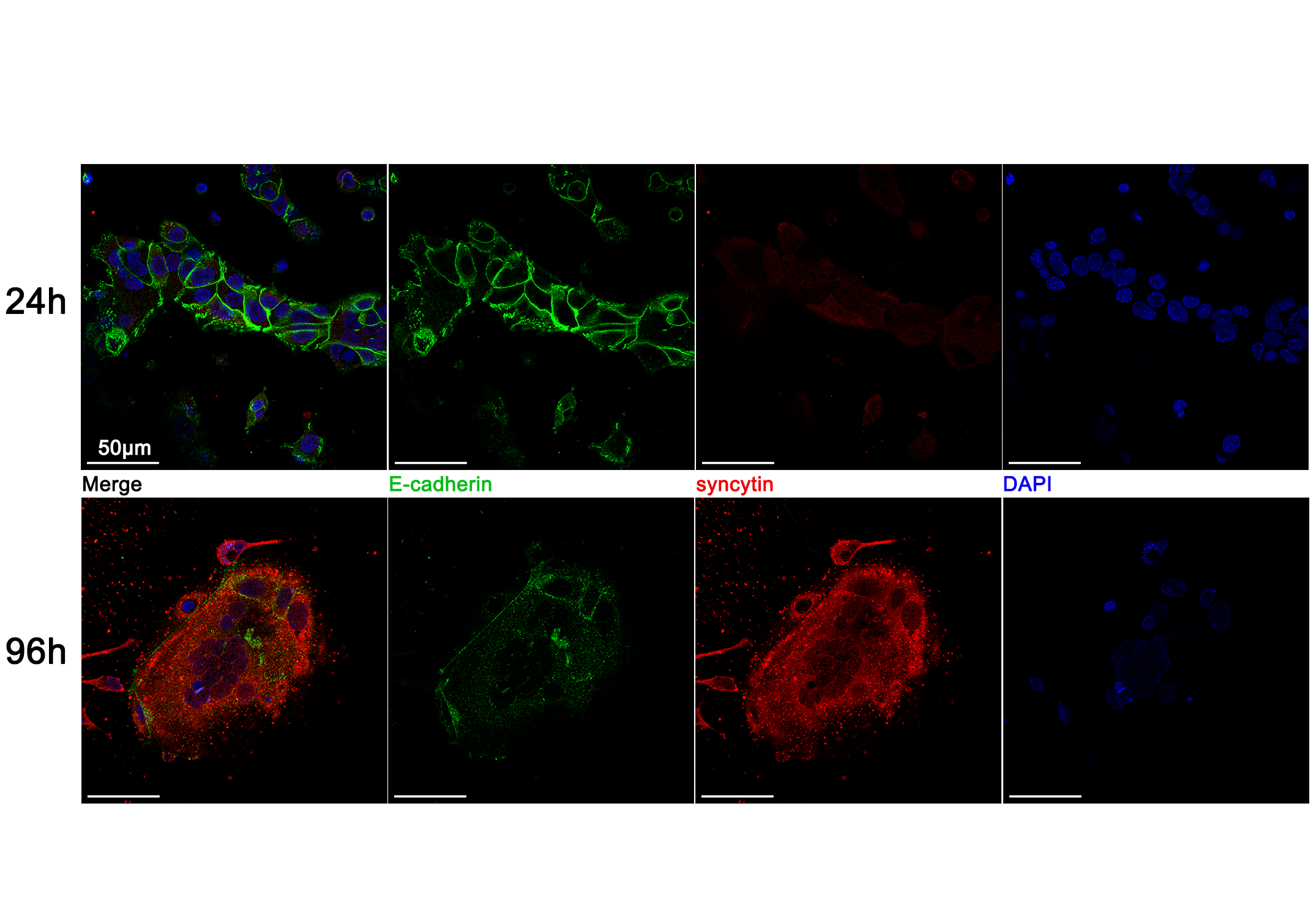

Supplement: Supplementary file 1 [file ijms-25-11534-s001.zip › Figure S4 - CTB and SCT ecadherin and syncytin.tif]

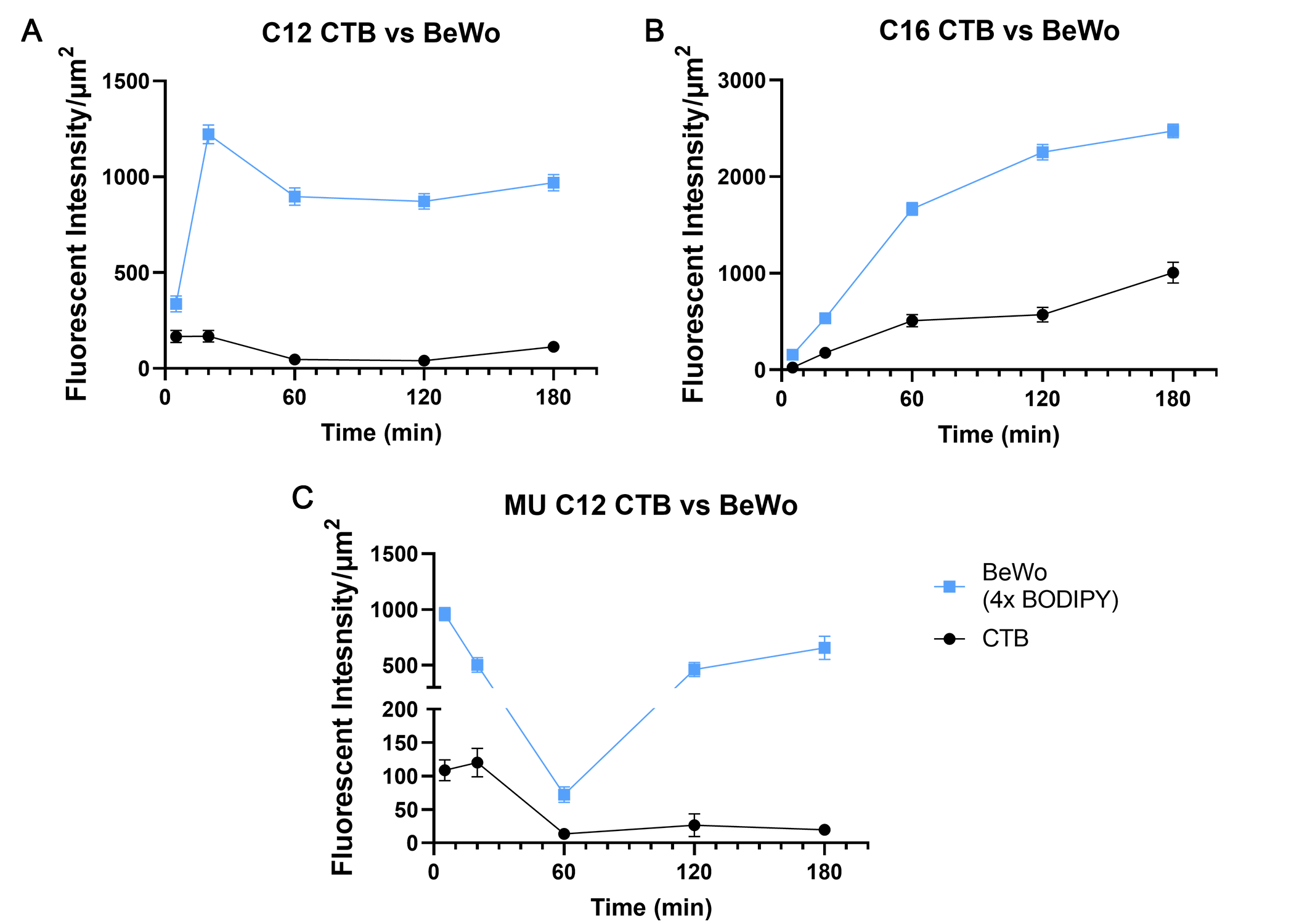

Supplement: Supplementary file 1 [file ijms-25-11534-s001.zip › Figure S5 - BeWo VS CTB FA Uptake.tif]

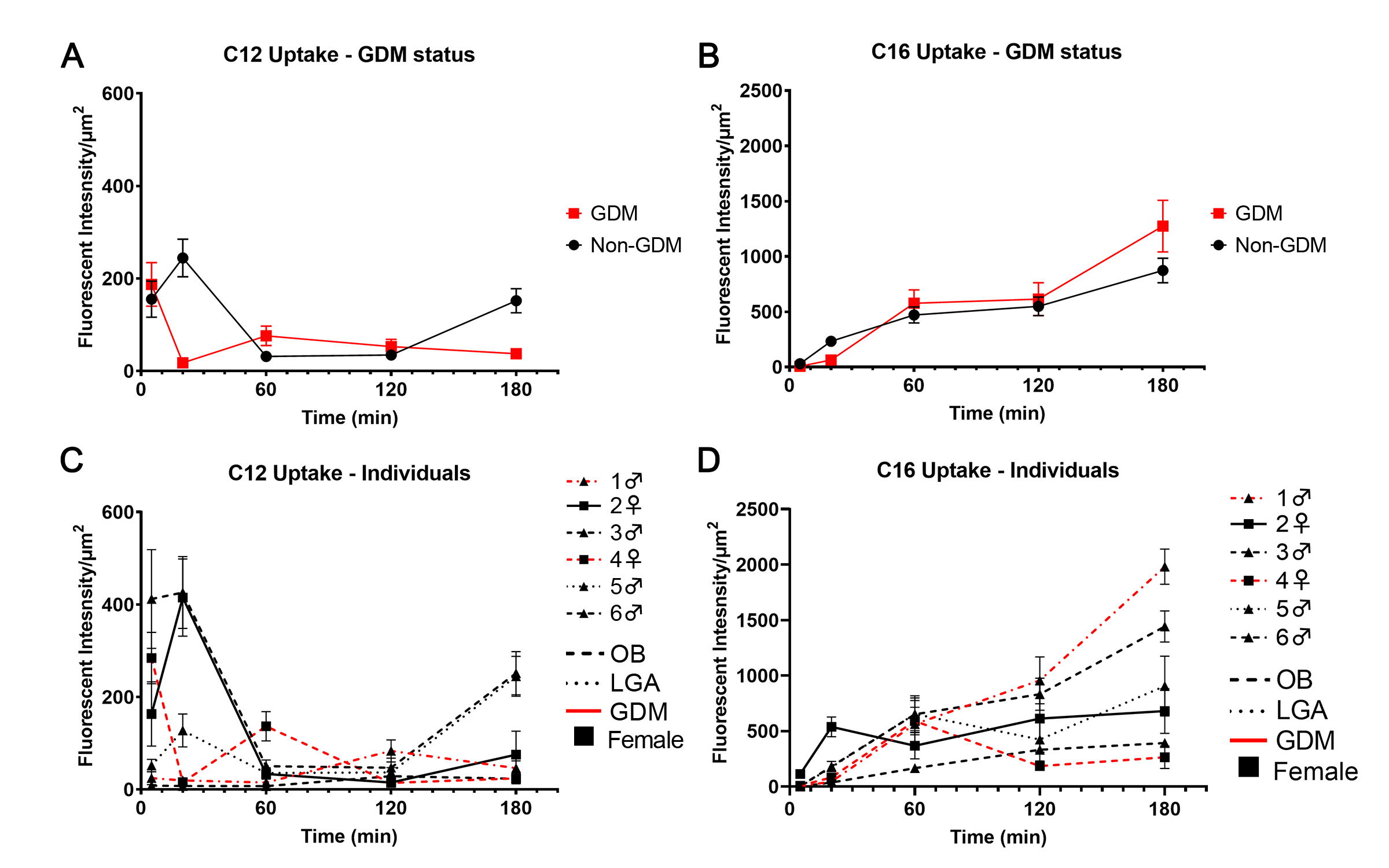

Supplement: Supplementary file 1 [file ijms-25-11534-s001.zip › Figure S6 - CTB FA uptake by patient.tif]

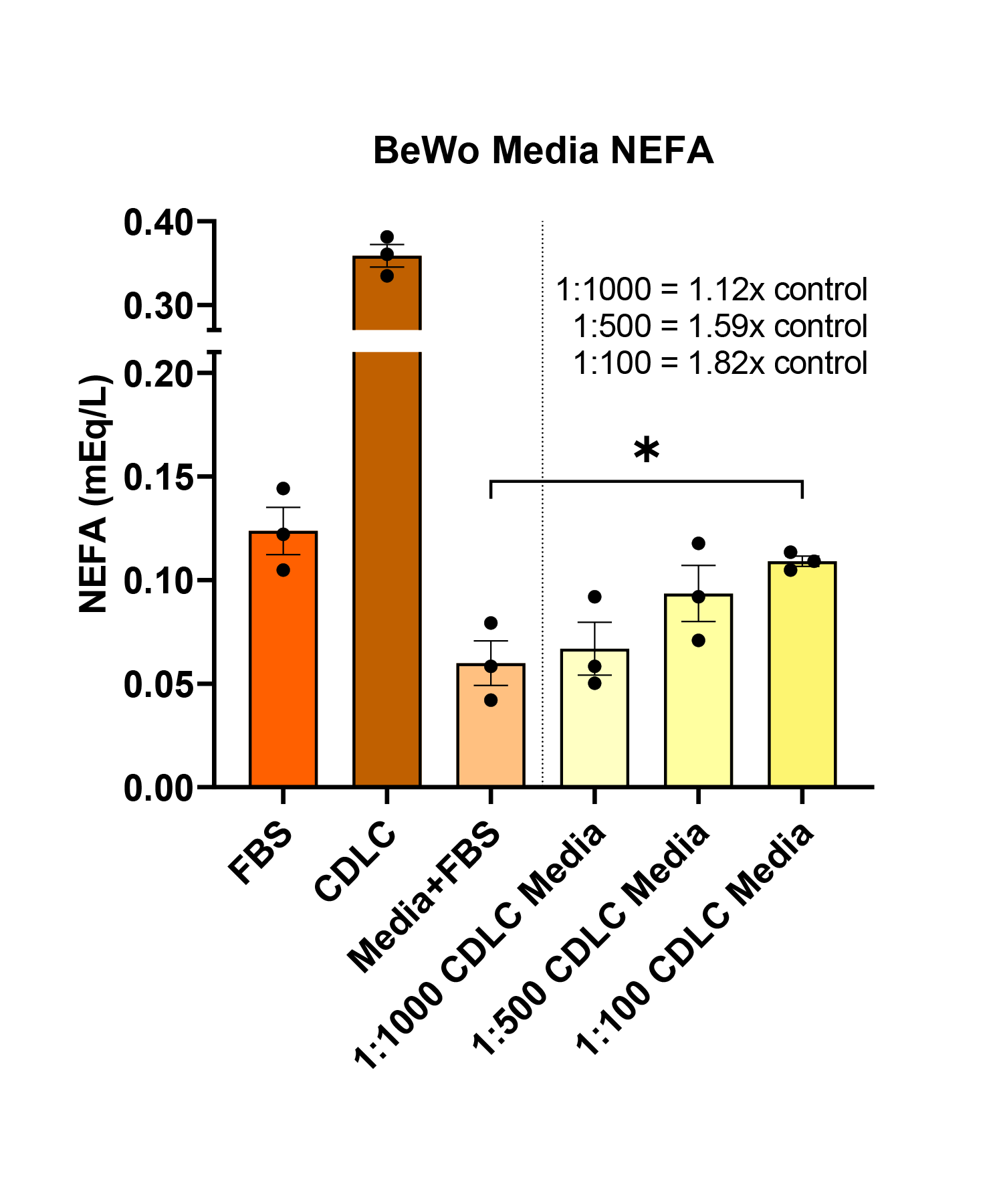

Supplement: Supplementary file 1 [file ijms-25-11534-s001.zip › Figure S7 - BeWo Media NEFA.tif]
